# Exceptional soft-tissue preservation of Jurassic *Vampyronassa rhodanica* provides new insights on the evolution and palaeoecology of vampyroteuthids

**DOI:** 10.1038/s41598-022-12269-3

**Published:** 2022-06-23

**Authors:** Alison J. Rowe, Isabelle Kruta, Neil H. Landman, Loïc Villier, Vincent Fernandez, Isabelle Rouget

**Affiliations:** 1 Sorbonne Université-MNHN-CNRS-CR2P, 4 Pl. Jussieu, 75005 Paris, France; 2 American Museum of Natural History, 200 Central Park West, New York, NY 10024 USA; 3 The European Synchrotron—ESRF, CS40220, 38043 Grenoble, France; 4 Imaging and Analysis Centre, The Natural History Museum, London, SW7 5BD UK

**Keywords:** Palaeontology, Palaeoecology

## Abstract

Although soft tissues of coleoid cephalopods record key evolutionary adaptations, they are rarely preserved in the fossil record. This prevents meaningful comparative analyses between extant and fossil forms, as well as the development of a relative timescale for morphological innovations. However, unique 3-D soft tissue preservation of *Vampyronassa rhodanica* (Vampyromorpha) from the Jurassic Lagerstätte of La Voulte-sur-Rhône (Ardèche, France) provides unparalleled opportunities for the observation of these tissues in the oldest likely relative of extant *Vampyroteuthis infernalis*. Synchrotron X-ray microtomography and reconstruction of *V. rhodanica* allowed, for the first time, a high-resolution re-examination of external and internal morphology, and comparison with other fossil and extant species, including *V. infernalis*. The new data obtained demonstrate that some key *V. infernalis* characters, such as its unique type of sucker attachment, were already present in Jurassic taxa. Nonetheless, compared with the extant form, which is considered to be an opportunistic detritivore and zooplanktivore, many characters in *V. rhodanica* indicate a pelagic predatory lifestyle. The contrast in trophic niches between the two taxa is consistent with the hypothesis that these forms diversified in continental shelf environments prior to the appearance of adaptations in the Oligocene leading to their modern deep-sea mode of life.

## Introduction

There is, to date, no undisputed phylogeny of the Cephalopoda that includes both extant and fossil taxa^1–4^. A scarcity of soft tissue preservation in fossil coleoids considerably restricts the number of characters available for comparison. Attempts at parsimony analyses highlight this significant imbalance, and the resulting bias limits the resolution and acceptance of existing phylogenetic trees^1,3^.

Material from Konservat-Lagerstätten provide a unique opportunity to study these otherwise lost soft tissue details^5–9^. The Jurassic Lagerstätten of La Voulte-sur-Rhône (Callovian, Ardèche, France) represents a bathyal ecosystem in an offshore environment with steep, fault-controlled bathymetric gradients^10,11^. This site is unique for its three-dimensional fossil preservation of photic and aphotic taxa^10^. The assemblage is diverse and consists of mostly arthropods^11–13^, as well as a few species of echinoderms^12,14^, bivalves, brachiopods, and fish^11^. Cephalopods constitute 10% of the biodiversity^15^ and approximately 20 specimens from the site have been assigned to the genus *Vampyronassa* by Fischer & Riou^16^.

Considerable attention has been paid to the position of Vampyromorpha^2,4,17–23^ as its only extant form, *Vampyroteuthis infernalis,* exhibits a mosaic of derived characters of both Octobrachia and Decabrachia^22^. It also has unique characters, including a well-developed gladius and two retractable filaments (arm pair II) in its arm crown which are not known in other extant cephalopods. After previously conflicting results regarding its affinity with Octobrachia or Decabrachia, the current consensus places *V. infernalis* as a basal member of the 8-armed Octobrachia^22–26^. *V. infernalis* is only known from deep-sea settings and is a detritivore, an opportunist consumer feeding on marine snow or zooplankton^27,28^.

Two fossil taxa have been assigned to the same family Vampyroteuthidae: the bathyal *Necroteuthis* Kretzoi 1942 (Palaeogene)^29^ known only from the gladius^22^, and the Jurassic *Vampyronassa rhodanica*^16^. Eight well-preserved specimens of *V. rhodanica* have been described from La Voulte-sur-Rhône^16^. These individuals retain the exceptional 3-D preservation associated with this site and therefore play an important role in understanding character evolution in Vampyroteuthidae and Vampyromorpha (Loligosepiina and Vampyromorphina)^22^.

Reanalysis of three *V. rhodanica* specimens using high-resolution imaging techniques provides a unique opportunity^30^ to observe soft tissue characters of this family. For the first time, we have a detailed reconstruction of external and internal morphology of *V. rhodanica,* with specific attention paid to the characters on the arm crown. These new anatomical data provide insights into the character states of the fossil form and were incorporated into a morphological phylogeny^1^. The current analysis supports the sister relationship between *V. rhodanica* and *V. infernalis*. Through the comparative morphology of the two vampyromorph taxa, as well as other extant and fossil forms, we suggest a palaeoecological reconstruction for *V. rhodanica* as a pelagic predator.

## Results

**Figure 1:**
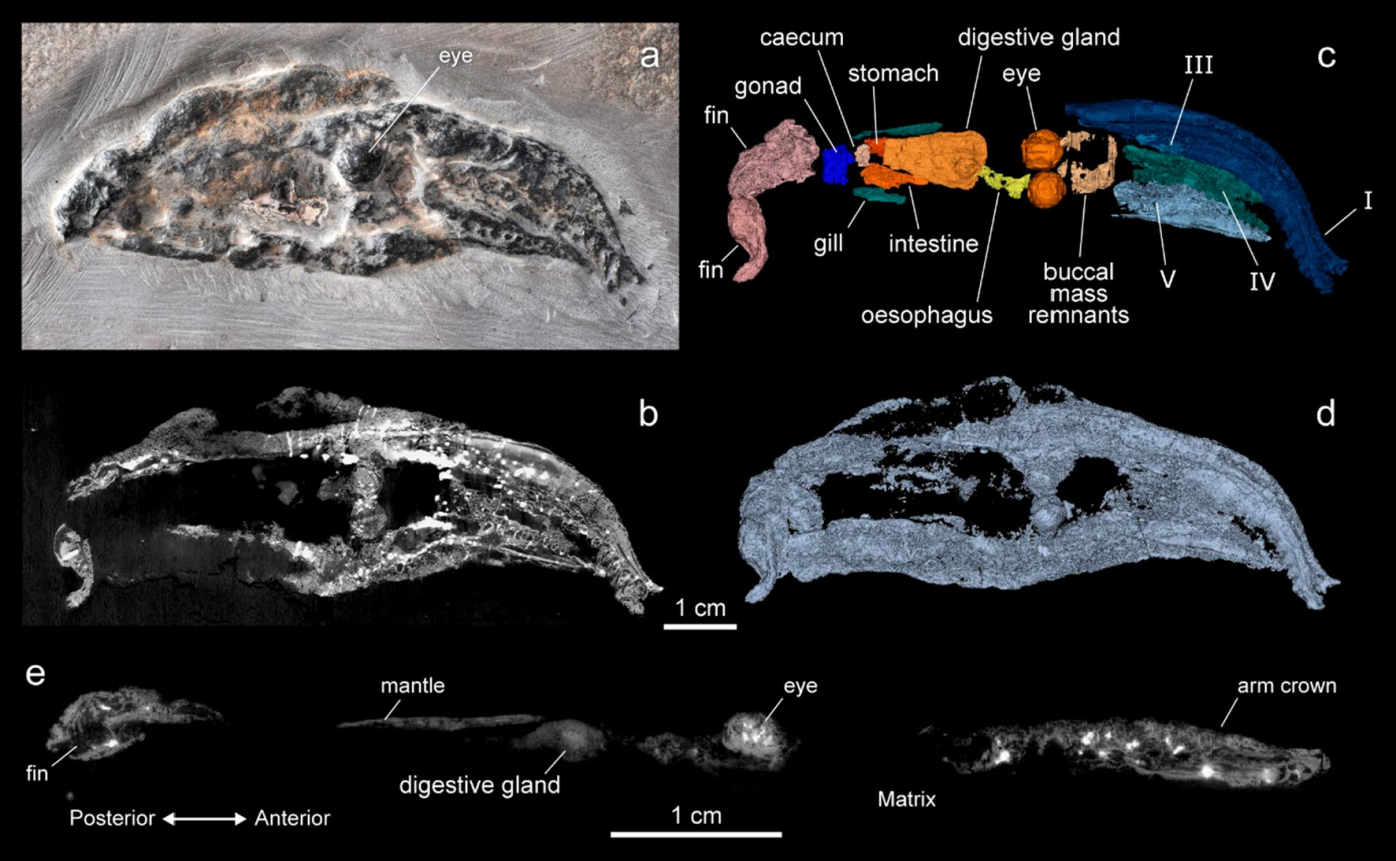
Images and reconstruction of the *V. rhodanica* holotype (MNHN.B.74247) acquired using PPC-SRµCT (voxel size: 12.64 µm), at the ESRF (Grenoble, France). (**a**) Photograph (P. Loubry, CR2P) of the specimen showing the 3-D preservation of the mineralised soft tissue. (**b**) PPC-SRµCT slice showing the contrast in the grey-scale image used to segment the specimen. This contrast results from the density variation among the various mineralised tissues. (**c**) 3D representation showing the arm crown (arm pair I, III, IV, and V), as well as other presumed internal elements (**d**) 3D reconstruction of the whole specimen (**e**) Sagittal slice showing the profile view.

Laboratory X- ray micro-computed tomography (µCT) and propagation phase contrast synchrotron X-ray micro computed tomography (PPC-SRµCT) data allowed for a reappraisal of the morphology in each of the three *Vampyronassa* specimens. (See Supplementary Information for a redescription of each specimen and details on the CT acquisitions.) Externally, *V. rhodanica* is elongate with an oviform body (Fig. 1). The three specimens studied range in overall length (posterior-most part of the mantle to the anterior-most tip of the arms) from ~ 94–103 mm (See Supplementary Information for additional individual measurements). The mantle appears posteriorly rounded in dorso-ventral view (MNHN.F.74244 (paratype)) and posteriorly tapered in lateral view (MNHN.F.74247 (holotype); MNHN.F.74243 (paratype)) (Supplementary Fig. 1). Two densely outlined elliptical shapes are interpreted as mineralized fin cartilage in the posterior mantle (MNHN.F.74244). A small fin protrudes on the dorso-lateral posterior section of MNHN.F.74247 and corresponds with previously described fin placement^16^.

The deep interconnecting velum, anteriorly extended funnel, and a cirri-like structure at the base of the dorsal arm described by Fischer & Riou^16^ could not be confirmed during segmentation. There was no evidence of an ink sac, retractable filaments, or modification of arm pair IV. Hectocotylization was not observed. Topographic differences on the dorsal, posterior section of the specimens suggest remnants of the inner shell (gladius), though the type of X-ray imaging used here does not allow us to provide details on its state.

The luminous organs described by Fischer & Riou^16^ could not be confirmed, though two dense, somewhat ovoid structures are located within the peripheral mantle tissue at the posterior-most area of the body in specimen MNHN.F.74244 (Supplementary Fig. 6). These dense structures are in a similar position to the luminous organs noted in the original description by Fischer & Riou^16^, though are 4–5 times larger in MNHN.F.74244. They are only observable in the tomographic image and do not appear in the other two specimens.

Head-mantle fusion is evident, and the head is approximately half as wide as the length of the mantle. Mantle tissue extends out from the body margins in MNHN.F.74247 and MNHN.F.74244 (Supplementary Fig. 1). This tissue is not preserved in a splayed position in MNHN.F.74243.

The eyes of each specimen are preserved, though their position is relative due to the amount of distortion in the body prior to mineralization. They are subcircular, have undergone various amounts of compaction, and range in diameter from 5 to 7 mm (Fig. 1).

**Figure 2:**
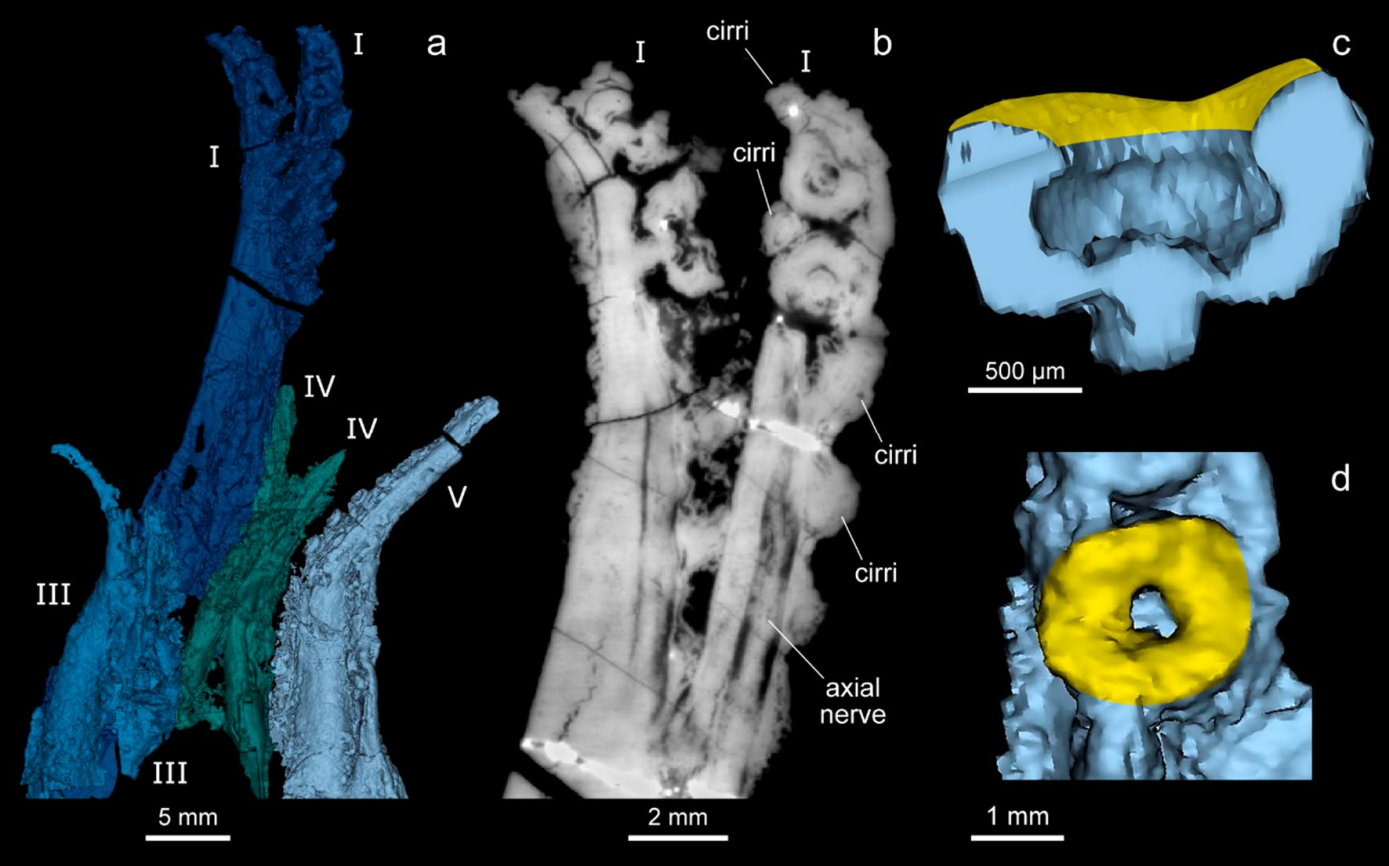
3D reconstruction and image of the arm crown, and a sample dorsal sucker of *V. rhodanica* (MNHN.B.74244). (**a**) Reconstruction of the arm crown (Mimics software) showing 8 arms, with the longer dorsal arm pair (arm pair I). (**b**) PPC-SRµCT slice of arm pair I (voxel size: 12.64 µm) showing the armature (2 uniserial suckers and flanking pairs of cirri) at the distal section, and the axial nerves. (**c,d**) 3D reconstruction of a dorsal sucker in profile and oral view respectively. The yellow colour reflects the location of the infundibulum.

Elements in the arm crowns are particularly well preserved. Each has 8 tapered arms and distinct axial nerves (Fig. 2). The dorsal pair (arm pair I) is approximately equivalent in length to the mantle, and roughly twice the length of the sessile arms (arm pairs III–V).

Each dorsal arm has two uniserial, radially symmetrical suckers, and paired cirri positioned on the distal section (Fig. 2a,b). These suckers on the dorsal arms are attached by a muscular elongated neck, which protrudes into the acetabulum (Fig. 2c). The angle of the infundibulum is shallow and oriented somewhat parallel to the arm (Fig. 2c). Four, possibly five pairs of primary cirri (Fig. 2a,b) precede the most proximal sucker, and then alternate with the suckers towards the tip. Contrary to the description by Fischer & Riou^16^, scans of the dorsal arms show no indication of proximal armature.

The remaining arms show very slight length variation with the arms preserved in ventral position (arm pair V) appearing marginally (a few millimetres) longer than the rest (arm pairs III and IV). It is not known if this is an artefact of preservation or a true character. Each of these arms has uniserial, radially symmetrical suckers and paired cirri (Supplementary Fig. 2). These features are present from the base to the tip of the arms. The suckers and cirri are similar in diameter, closely positioned and taper distally. Some suckers appear to be encircled by ovoid depressions in the peripheral tissue (Supplementary Fig. 3), though there is no evidence to indicate that these correspond with toothed sucker rings^31,32^ found in some Decabrachia^2, 33–35^. This detail appears in each of the *V. rhodanica* specimens, though it is not present on every sucker. The position of the depressions on the outer margins is not consistent with the feature being a remnant of the internally placed sucker rings in some Decabrachia^35^, and there is no evidence of this character being present^32^. Similar looking tissue is visible elsewhere on the arms, including on the profile views of the cirri and suckers (Fig. 2b). Without further evidence to suggest otherwise, we suggest these depressions are a manifestation of degraded epithelium.

**Figure 3:**
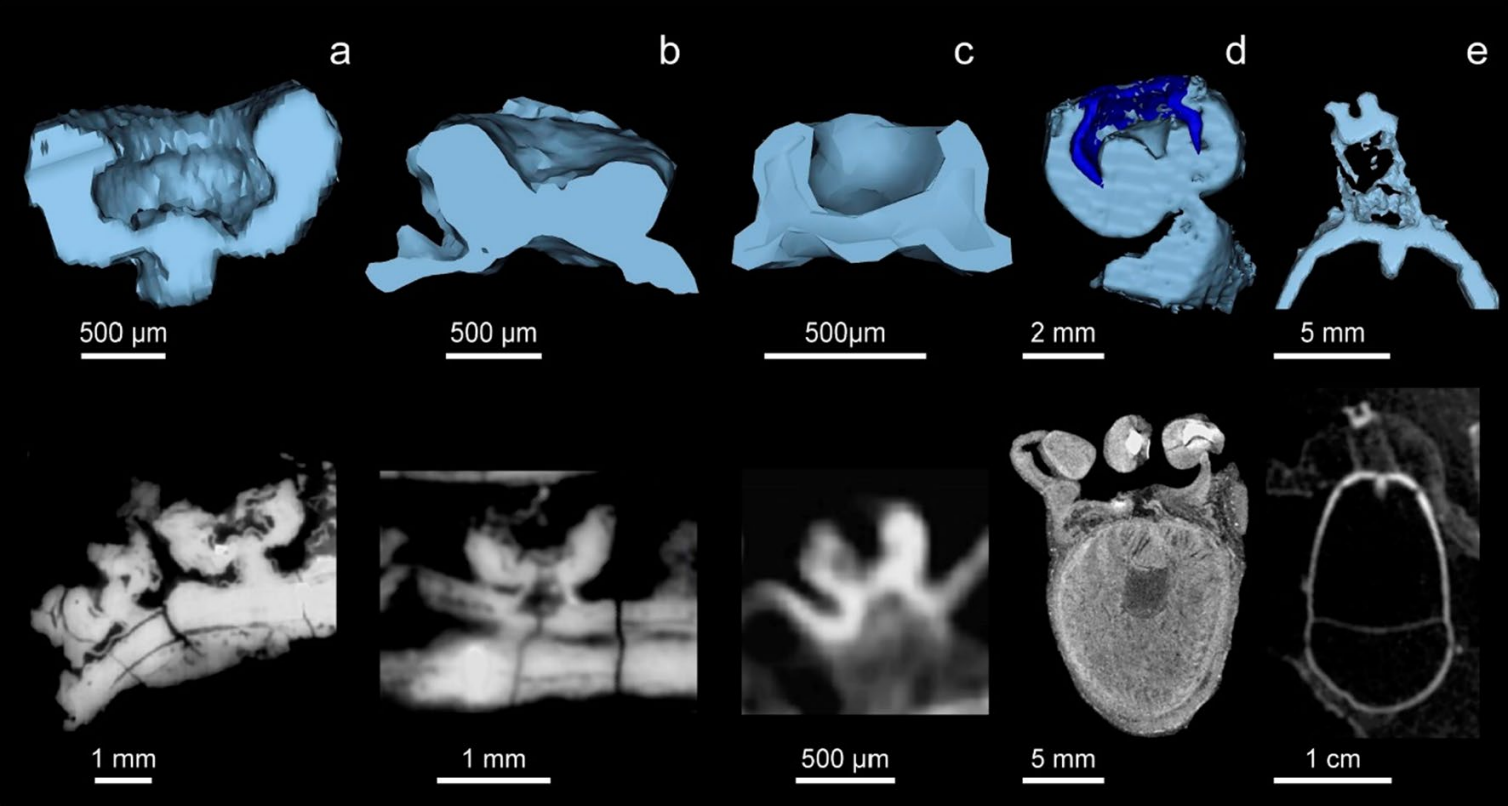
3D reconstructions (top) and virtual slices (bottom) of sucker profiles. (**a,b**) Dorsal, and sessile sucker profiles of *V. rhodanica* MNHN.B.74244 respectively (PPC-SRµCT, ESRF). (**c**) *V. infernalis* (AMNH IZC 361496) (Vampyromorpha) (**d**) Commercial *Loligo* (Decabrachia) sample (µCT, AST-RX). (**e**) *Grimpoteuthis* (ZMB 240160) (Octobrachia) CT data from Ziegler et al.^58^ and reconstructed for this study. Extant material (**d,e**) was stained prior to scanning.

All the suckers in the arm crown display a conical, *Vampyroteuthis*-like attachment (Fig. 3c, and Supplementary Fig. 4) and do not show a clear attachment to the arm muscle. There is slight variation between the sucker stalks on the dorsal arms and the rest of the arms in the arm crown (Fig. 3a,b respectively). The stalks connecting the two suckers to the dorsal arms are slightly longer and narrower than the more compact, triangular-shaped attachments that connect the suckers along the length of the sessile arms.

These new morphological data were incorporated into the existing phylogenetic matrix of Sutton et al.^3^ that was modified by Kruta et al.^1^. The analyses returned 34 trees, and the data clarified four characters that were previously unresolved (#89-#92: Sucker symmetry; presence of sucker stalks; sucker lining, and the presence of proximal suckers, respectively. The consensus tree has the same topology as seen in Kruta et al.^1^, with each of the state changes at node 134. This supports the hypothesis that *V. rhodanica* and *V. infernalis* are more closely related to each other than they are to any other taxon in this analysis (Supplementary Information Fig. 5). A radially symmetrical sucker state (character #89) was already known in *V. infernalis*. Our observations confirmed that this state is also reflected in *V. rhodanica*.

A character state was added to #91 to best reflect the shape of the stalks attached to the arm muscles. In Kruta et al.^1^, this state is described as either a “conical pillar with base and neck” (state 0) consistent with modern Decabrachia, or a “cylinder” (state 1) consistent with modern Octobrachia. The shape of the sucker stalk in *V. rhodanica* resembles the attachment seen in modern *V. infernalis* (Fig. 3b,c)*.* As such, character #91 was amended, adding a “base and plug”^34^ state (state 2) to reflect the states outlined in Young & Vecchione^34^. For character #92^1^, *V. rhodanica* showed no evidence of either a “horny” (state 1) or “cuticular [sucker] ring” (state 2) (see Fuchs et al.^35^ for a full explanation of this character) on the inner lining of the sucker. In this study, both *V. infernalis* and *V. rhodanica* were coded as (state 0), to reflect the lack of this sucker lining. According to our results, (state 1) was common in Decabrachian taxa, and *V. infernalis* and *V. rhodanica* (state 2) were nestled within Octobrachia where this character was typically ambiguous. The type of muscular connection within the sucker attachment (character #90) proved to be of most interest. In *V. infernalis,* this state (state 2) is “present but not clearly attached to the arm muscles”^34^. This had previously been considered an autapomorphic character in *V. infernalis* though it is also present in *V. rhodanica*. It is, in fact, a synapomorphy of node 134 that links the two species and increases the robustness of the node.

## Discussion

In their original description of *V. rhodanica*, Fischer & Riou^16^ determined that the previously undescribed genus was a Jurassic relative of *V. infernalis*. This assignment was based on the configuration of the arm crown and armature, fin type, presence of luminous organs, lateral eyes, and the absence of an ink sac. Assuming this assignment is correct, then *V. rhodanica* is a member of the suborder Vampyromorphina, which includes the family Vampyroteuthidae^22,29^.

Reappraisal of the anatomy shows that *V. rhodanica* and *V. infernalis* both have 8 arms and uniserial suckers flanked by cirri. They both possess *V. infernalis*-like sucker attachments^34,36^, which are broader at the base and taper up to a radially symmetrical sucker.

Both species have distinctly modified arms though the morphology differs in each. *V. infernalis*, has retractable filaments in the position of arm pair II^27,33,34^, though there is no evidence of these appendages in *V. rhodanica.* Instead, the species has elongate dorsal arms (arm pair I) with a unique configuration of suckers and cirri on the distal section.

**Figure 4:**
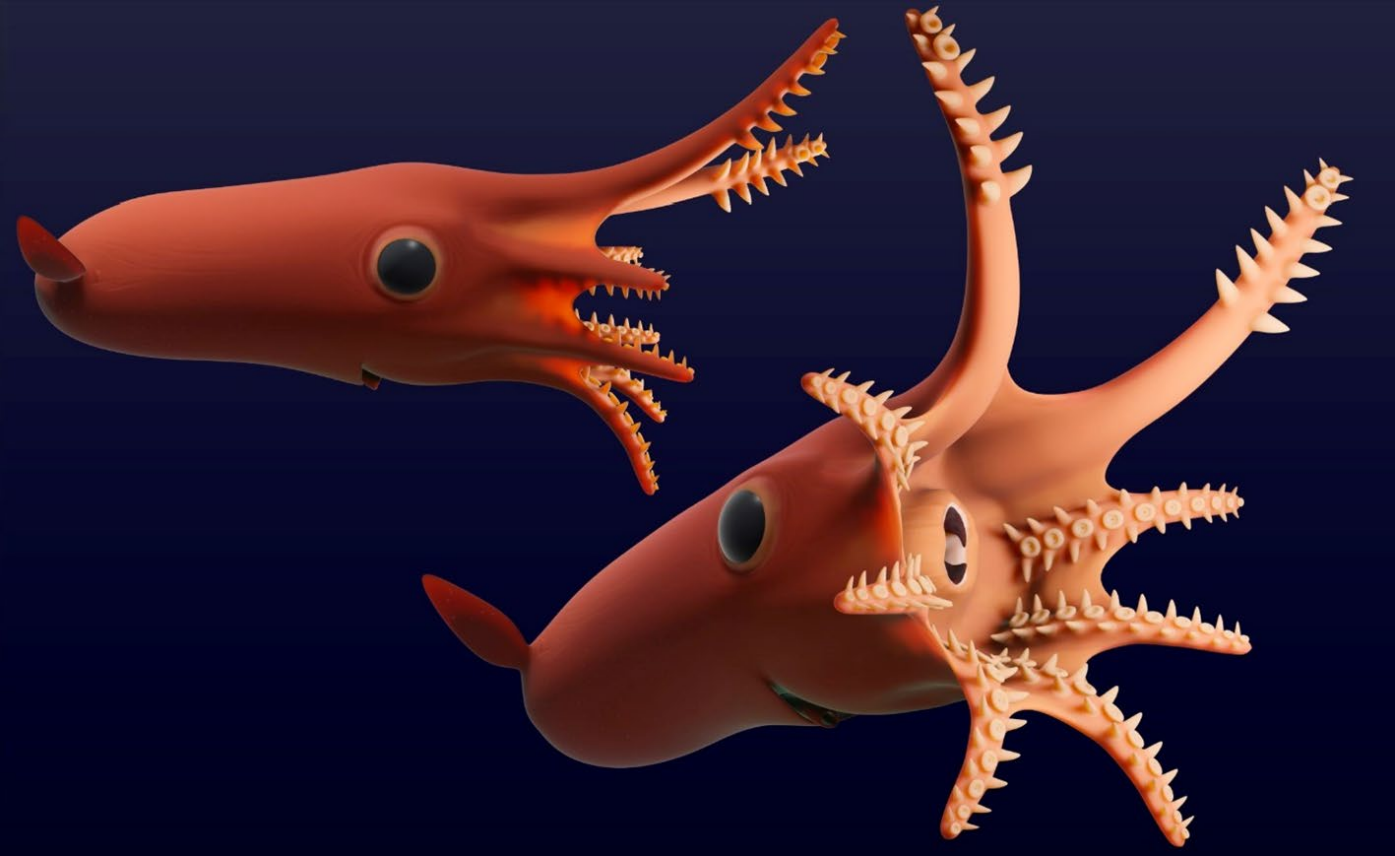
Hypothesised reconstruction of *V. rhodanica* based on the data from this study (A. Lethiers, CR2P). The scale is based on measurements from the holotype (MNHN.B.74247) and the arm crown is completed using dimensions from MNHN.B.74244.

The suckers and cirri of *V. rhodanica* are more numerous than those of *V. infernalis*^27,37^. They are also more closely positioned. Proportionally, the suckers of both species have a consistent ratio to mantle length^37^, though the diameter of the cirri and infundibulum are greater in *V. rhodanica*. The *V. infernalis*-like attachment^1,3,34^ is present in both species, though in *V. rhodanica*, the distal part of the neck protrudes into the acetabular cavity. Of note, the sucker stalks on the dorsal arms of *V. rhodanica* are more elongate than those on the other arms (Figs. 2b,c, and 3a,b). This variation in suckers and their attachments suggests a specialized function between the dorsal and sessile appendages. On the longer dorsal arms, the larger sucker diameter, and more elongate stalks (Figs. 2b and 4) indicate the potential for increased mobility over their extant relatives, and possibly facilitated additional manipulation and prey capture capability.

In addition to the arm crown specialization, *V. rhodanica* has a more streamlined shape than *V. infernalis*, which is caused by a proportionally narrower head. Their muscular body is narrower and more elongate than the gelatinous *V. infernalis*^16,27,37^ suggesting a higher energy locomotory style. This is consistent with increased predation relative to the modern form. Observations in this study support many assertions of Fischer & Riou^16^ about the characters in *V. rhodanica*, though the presence of luminous organs cannot be confirmed. Rather than luminous organs much larger than those present in the deep-sea, extant *V. infernalis*, it is possible that these structures represent displaced cartilage prior to fossilization (Supplementary Fig. 6).

Two other genera from the La Voulte-sur-Rhône locality, *Gramadella* and *Proteroctopus* are, like *V. rhodanica*, considered to be *Incertae sedis* Vampyromorpha^22^. All three share morphological similarities that include an elongated mantle fused with the head, and a longer dorsal arm pair with armature on the distal ends^1,16,22,38^. Neither the second nor fourth arm pair have been modified. Each has one pair of fins. In *Gramadella,* the fins are lateral and skirt-like^16,38^. In *V. rhodanica* and *Proteroctopus* these fins are located posteriorly^1,16^.

*V. rhodanica* shows the greatest length variation between the dorsal and sessile arms (Fig. 4), though proportionally, *Gramadella,* and *Proteroctopus* have longer dorsal arms^1,31^. Fischer & Riou^31^ and Kruta et al.^1^ described biserial suckers in their descriptions of *Gramadella*, and *Proteroctopus,* respectively. In *Proteroctopus*, these suckers have a proportionally smaller diameter than the uniserial row in *V. rhodanica,* and do not exhibit the same tapered pattern.

None of these specimens shows evidence of an ink sac, though it is present in contemporaneous genera from the same assemblage (*Mastigophora, Rhomboteuthis* and *Romaniteuthis*)^8,16^. That this character occurs only in some taxa from the same assemblage suggests variation in ecology, possibly associated with the steep, bathymetric relief in the La Voulte-sur-Rhône paleoenvironment^11^. The mosaic of characters found within the coleoid taxa at La Voulte-sur-Rhône suggests that Mesozoic vampyromorphs co-occurred in different ecological niches during the mid-Jurassic.

Today, extant *V. infernalis* is uniquely adapted to a low-energy, deep-sea mode of life^27–29,39^, though the timing of character acquisition and progression of this ecology is unclear^24^. It is hypothesised that the vampyromorph *Necroteuthis* Kretzoi 1942 was already exploiting this niche by the Oligocene^29^, and that the initial shift to offshore environments was possibly driven by onshore competition^24,29^. The data obtained here suggests that *V. rhodanica*, the purportedly oldest-known genus of the Vampyromorphina group, was an active predator following a pelagic mode of life.

Indeed, several anatomical details, mainly found in the brachial crown, seem to support this hypothesis. Though we cannot directly compare functionality of the arm crown elements with other Jurassic taxa, we can infer function based on observation in modern forms. In Octopoda, the sister group to Vampyromorpha, suckers are attached to the arm by a cylindrical layer of muscle, encircling oblique musculature^40,41^, that connects the arm musculature and the lateral margin of the acetabulum^34,40–42^. This facilitates a variety of functions including locomotion, manipulation, and prey retention^43^. The sucker attaches by flattening the infundibulum against the surface and then the encircling epithelium creates a watertight seal^36,40–45^. Contraction of the radial acetabular muscles provides the pressure differential required to create the suction force^43,44,46^.

The stalked sucker attachments^2,34^ of decabrachians (Fig. 3d, and Supplementary Fig. 4) are muscular^35^ and connect the musculature of the arm with the base of the sucker, forming part of the acetabulum^33,34^. Tension on the sucker stretches this muscular attachment, which pulls locally on the acetabular base. This facilitates a greater pressure differential inside the sucker, allowing the teeth on the sucker ring to maintain the hold^47^.

Extant *V. infernalis* lack decabrachian-like stalks^2,18^ and the neck of the attachment joins to the base of the acetabulum (Fig. 3c, and Supplementary Fig. 4), rather than being inserted into it^18^. The infundibulum is not distinct, and the suckers do not provide strong suction^27^. Instead, suckers function by secreting mucus to coat detritus—marine snow captured by retractable filaments—which is then moved to the mouth by cirri^7,27^.

A mosaic of these characters is present in *V. rhodanica* (Fig. 3a,b), therefore, suggesting their potential for increased attachment and hold on prey over extant *V. infernalis*. These include a larger infundibular diameter, a neck attachment integrated with the acetabular muscles, and the elongated stalks of the dorsal suckers.

Additionally, the paired, filamentous cirri observed in extant cirrates^48^ are present in *V. rhodanica* (Fig. 4, and Supplementary Fig. 2). In extant forms they are understood to have a sensory function and are used in the detection and capture of prey^48^. In *V. infernalis*, they serve to transport the food proximally along the arms to the mouth^27^. The greater diameters of cirri, and placement along the entire arm in *V. rhodanica* (Fig. 4), suggests an increased sensory function in these fossil forms.

The shape of the arms also contributes to the suction potential^49^ in coleoids. Functional analysis in Octopoda highlights a positive correlation between distal tapering of the arms and their flexibility. A tapered, flexible arm facilitates more precise adhesion than a cylindrical-shaped one and requires a greater force for sucker detachment^49^. Suckers detach sequentially, rather than the more simultaneous release observed in models of arms with less taper variation. The tapered diameter of the suckers, like those seen on the sessile arms of *V. rhodanica*, potentially facilitated this kind of sequential detachment^49^ allowing them more adherence force and flexibility. Though *V. rhodanica* has just two suckers on the distal tips of their dorsal arms, the most distal is marginally smaller in diameter than the proximal one. On the dorsal arms, this tapering is observed in conjunction with a well-developed axial nerve cord (Fig. 2b). In extant forms, the nerve cord facilitates complex motor functions^42^. The combination of these characters in *V. rhodanica* suggests their arms had increased potential to be actively used in prey capture^50^ over extant *V. infernalis*.

Though arm crown characters offer insight on the ecology of *V. rhodanica*, in fossil coleoid phylogenies only a few characters are based on the suckers^1, 3^. Two studies that have attempted to create a phylogeny using morphological characters that include both fossil and extant taxa return *V. rhodanica* and *V. infernalis* as sister taxa^1,3^. These matrices are, by necessity, heavily influenced by the gladius^51^ and more than 50% of the characters are based on this feature^1,3^. Indeed, the authors^1^ note that the lack of gladius data for some fossil forms, including *V. rhodanica,* creates an inherent bias in the phylogenetic matrix. Fischer & Riou^16^ suggested that *V. rhodanica* and *V. infernalis* are related on the basis of the observable morphological characters in the family Vampyroteuthidae, though without morphological information on the gladius, a recent systematic synthesis of fossil Octobrachia^22^ positioned *V. rhodanica* as Vampyromorpha *Incertae sedis*.

X-ray CT analysis in this study did not allow a reconstruction of the gladius. Nevertheless, it does provide new data on soft tissues, and permits comparisons between extant and fossil taxa. Specifically, we can add distinct states to 4 of the 132 characters in the existing phylogenetic matrix from Sutton et al.^3^ that was modified and used in Kruta et al.^1^. These four characters (#89–#92) represent the suckers, and sucker attachments. Detailed examination revealed that the sessile and dorsal arms have the *Vampyroteuthis*-like attachment. In the dorsal arms, this is more elongated, though it cannot be considered pedunculate like those seen in modern decabrachians. Indeed, the attachment type (plug and base^34^) is the same, only the length varies. As previously discussed, this variation may have functional implications.

When updated with these new data, the matrix from this study returns the same topology seen in Kruta et al.^1^ that supports the positioning of *V. rhodanica* and *V. infernalis* as sister taxa. Further, it strengthens their relationship as they both share a sucker attachment that is not clearly attached to the arm muscles, a state that was previously considered autapomorphic in *V. infernalis*. However, it is important to note that no additional characters were added for the gladius, which is the cornerstone of coleoid systematics^52^. Indeed, just 29 of the 132 matrix characters can so far be coded for *V. rhodanica*, with only 9 of these relating to the 74 states of the gladius.

Assuming the phylogenetic work so far is correct, then both species belong to the family Vampyromorphina, and are joined by the Oligocene fossil *Necroteuthis hungarica*^29^. While the lack of gladius characters precludes a full phylogenetic understanding of this group, preservation and observation of the soft tissues allow us to infer information regarding palaeobiology.

The data obtained in this study demonstrates that the characters observed in *V. infernalis,* including the sucker attachments and lack of ink sac, were present in Jurassic Vampyromorpha. Comparative anatomy of *V. rhodanica* and extant *V. infernalis* revealed that the fossil taxon displayed more morphological variation and were more diversified than previously understood. The assemblage of characters observed in *V. rhodanica* are consistent with a pelagic predatory lifestyle and corroborate the likelihood of a distinctly different ecological niche. These findings support the hypothesis that a shift towards a deep-sea environment occurred prior to the Oligocene^5,29^.

## Methods and materials

### Materials

Three fossil specimens of *V. rhodanica* (holotype MNHN.B.74247, and two paratypes MNHN.B.74244 and MNHN.B.74243) from La Voulte-sur-Rhône provided the basis for this study. These samples are reposited in the paleontological collections of the Muséum National d'Histoire Naturelle (Paris, France). Some anatomical features in these three specimens are absent due to distortion and tissue loss prior to mineralization, as well as during the preparatory process. Each specimen exhibits varying levels of deformation. Despite this, each has retained exceptional 3-D morphology.

This exceptional preservation was a result of soft tissue replacement during a sequence of mineralization phases^53^. Ordinarily, calcium carbonate precipitates in marine environments^54,55^ but at La Voulte-sur-Rhône, rapid post-depositional microbial activity in the low oxygen setting reduced pH, leading to authigenic precipitation of iron-rich minerals^53,55–57^. Mineralization was localized in the organism, and the organs and tissues were potentially replaced by different mineral phases^54, 55^. Analyses of marine arthropods mineralized at La Voulte-sur-Rhône show that muscle tissue is replaced by fluorapatite and pyrite (and related sulphides)^53^. The same is assumed here for *V. rhodanica*. It is the density of these fossilized tissues that provides the grey-scale contrast observed in the tomographic imagery.

A subsequent reset of the pH reverted to calcite precipitating conditions^53,56,57^ and allowed the preservation of fine morphological detail^55^. In some instances, calcium carbonate concretions formed around the specimens^53^.

CT data of extant forms (*V. infernalis,* AMNH IZC 361496 and YPM IZ 018297.GP), and *Grimpoteuthis* and *Sepia* from MorphoBank project (#3107)^58^ were also analysed for comparison.

### Microtomography

The three *V. rhodanica* fossils were initially imaged using µCT at the AST-RX platform at the MNHN and then using PPC-SRµCT at the European Synchrotron Radiation Facility Synchrotron (ESRF, ID 19 beamline, Grenoble, France). PPC-SRµCT data have a voxel size of 12.64 µm (MHNH.B.74247, MHNH.B.74243 and MHNH.B.74244); AST-RX platform µCT data have a voxel size of 88.60 µm (MHNH.B.74244). Specimens of *V. infernalis* were analysed using µCT at the Microscopy and Imaging Facility of the American Museum of Natural History (New York, USA). The voxel size for each specimen analysed was 38.40 µm for AMNH IZC 361496, and 18.25 µm for YPM IZ 018297.GP. See Supplementary material for microtomography details.

Final CT data were reduced in size using ImageJ software (cropping and size reduction by binning 2 × 2 × 2), and then segmented using Mimics software (Materialise NV, Belgium, Version 21.0). The contrasting densities of the mineralized soft tissues were utilized to identify anatomical features for segmentation. Morphological reconstructions were carried out for the three *V. rhodanica* specimens incorporating all possible internal and external soft tissues. A full reconstruction of *V. infernalis* was carried out on AMNH IZC 361496. Some suckers in YPM IZ 018297.GP had more clearly defined boundaries and these were integrated into the analysis to augment the data gathered from AMNH IZC 361496.

### Phylogenetic analysis

New character state data obtained from the segmentation of *V. rhodanica* were incorporated into the phylogenetic matrix from Kruta et al.^1^. This matrix is built on 132 characters that describe morphological states of fossil and extant forms. More than 50% of these are based on the gladius. One new state was added to character 91 to reflect State 9 in Young and Vecchione^34^. Characters described by Fischer & Riou^16^ that were not able to be observed by segmentation in this study, remain unchanged in the matrix. The dataset was analysed using TNT v.1.1^59^ with implied weighting (concavity constant of K = 3). The monophyly of the decabrachians was constrained as in Sutton et al.^3^.

### Methods

Comparative studies were also conducted with fossil specimens, and descriptions and images were taken from the literature. Most of these character comparisons focussed on the arm crown, though the fins and ink sac were also included. The resulting fossil sample comprised three *Incertae sedis* Vampyromorpha, *Gramadella, Proteroctopus,* and *V. rhodanica*, as well as the loligosepiid *Mastigophora*.

Measurements for all specimens were collected using parameters outlined in Fig. 3 of Nixon 2011^36^. The mantle length was taken from the central lateral line of the eye to the most posterior part of the body. Arm length was taken from the central lateral line of the eye to the most anterior tip. As none of the specimens is preserved in anatomical position, all measurements are composite. Measurements followed the natural line of the form where possible.

The ratio calculations performed on these measurements were defined in Pickford^37^ and are detailed in Supplementary Information. Pickford^37^ provided a comprehensive account of measurements and ratios for *V. infernalis*, and the mean values provided were utilized for comparative analyses. The same ratios were used to calculate proportions in the fossil and extant forms where possible. Pickford^37^ noted two equations to determine the length of longest arm: the arm length index and the mantle length index. The arm length index was used for the *V. infernalis* specimens, and therefore was used in this study. We used this formula also. Where possible, the various indices were calculated twice; once using measurements from the dorsal arms, and the others for the measurements taken from the shorter sessile arms. Ratios for elements within the suckers were not provided in Pickford^37^ so an adaptation was used (Supplementary Information). From this, we calculated proportional values for the infundibular diameter, cirri diameter, and the acetabular cavity.

## Supplementary Information

Supplementary Information.
