# Supplementary material for: Exceptional soft-tissue preservation of Jurassic Vampyronassa rhodanica provides new insights on the evolution and palaeoecology of vampyroteuthids
Source: Sci Rep. 2022 Jun 23;12:8292. doi: 10.1038/s41598-022-12269-3 (PMC9225997; doi:10.1038/s41598-022-12269-3)
Supplement: Supplementary file 1 — Supplementary Information. [file 41598_2022_12269_MOESM1_ESM.docx]

**Supplementary Information for:**

**Exceptional soft-tissue preservation of Jurassic *Vampyronassa rhodanica* provides new insights on the evolution and palaeoecology of vampyroteuthids**

Alison J. Rowe^1^, Isabelle Kruta^1^, Neil H. Landman^2^, Loïc Villier^1^, Vincent Fernandez^3,4^, Isabelle Rouget^1^

^1^Sorbonne Université-MNHN-CNRS-CR2P, 4 Pl. Jussieu, 75005 Paris, France. ^2^American Museum of Natural History, 200 Central Park West, New York, NY 10024, United States ^3^ The European Synchrotron—ESRF, CS40220, 38043 Grenoble, France, ^4^ Imaging and Analysis Centre, the Natural History Museum, SW7 5BD, London, United Kingdom

1. **Redescription of *V. rhodanica* Specimens**

**Holotype: MNHN.B.74247**

The holotype of *V. rhodanica,* MNHN.B.74247 (Fig.1) is fossilized in lateral view. It has an oviform, tapered mantle, and measures ~43 mm from the central line of the eye to the posterior tip.

Two small posterior fins are evident at the posterior of the mantle. One is displayed as a small projection extending outwards from the body. It is not possible to determine an exact shape, though given the positioning of the anterior-most part, it appears to have been attached only at the posterior section. The other fin is less apparent and seems to have been folded against the body prior to mineralization of the specimen. The associated fin cartilage is observed as two distorted ovoid shapes, with internal striated, curved strips that are denser than the surrounding tissue. The posterior section of the body has clearly been twisted as the two areas of fin cartilage are no longer on the original plane.

With the benefit of tomographic imaging, it is possible to identify a sinuous strip of soft tissue projecting along the margin that has been preserved in a dorsal position. This is denser than the surrounding matrix and has a somewhat mottled appearance. There is a distinct boundary on the outer edge of the tissue. It appears just in front of the right eye, extends almost all the way to the posterior, and tapers at both ends. There is no obvious corresponding counterpart splayed out on the other margin to indicate. Without further evidence it is not possible to determine the exact nature of this tissue, though here, we suggest it is splayed mantle tissue.

Elements of the digestive, respiratory, and reproductive systems are preserved, though the considerable compression and missing parts of the body makes it challenging to identify them with certainty. Additionally, despite topographic changes in the mantle indicative of a gladius, the X-ray imaging does not allow us to provide details on its presence or state. The holotype shows no evidence of an ink sac.

As in each of the specimens redescribed here, the head is short and represents the widest part of the body (~23 mm in the holotype). This appears to be fused to the mantle as no neck is evident. The eyes (~5-6 mm in diameter) are offset and preserved on different planes, reflecting some rotation of the head prior to mineralization.

MNHN.B.74247 retains 8 non-retractile (sessile) arms preserved in lateral view, the flesh of which tapers slightly toward the tip. Arm pair I are most dorsally situated in the arm crown and, as with the two other specimens, are the longest. Arm pair I (~49-51 mm) has armature only on the distal section of the arm. This is comprised of laterally paired cirri (no more than 2 mm in diameter as the base) and two, uniserial suckers (~2 mm in diameter) with radial symmetry. These have *Vampyroteuthis*-like attachments though are slightly more elongate that those on arms pairs III-V. There is no evidence of sucker rings.

Four, or perhaps five pairs of cirri precede the most proximal sucker, and an additional pair are positioned laterally between the proximal and distal sucker. No penultimate anterior pair of cirri are evident on the holotype, though a comparison of all three specimens (MNHN.B.74247; MNHN.B.74244; MNHN.B.74243) indicates that cirri are present in this location.

Arm pairs III to V range in size from approximately ~31 – 34 mm in composite length. They feature a single row of suckers on the oral surface which are present from the base to the tips of the arms. These taper distally and range in diameter from ~2 mm proximally to ~1 mm at the tip. Paired cirri are arranged on either side of the suckers (~13) along each arm. They have a maximum diameter of 2mm and taper in size toward the tip of the arms. Axial nerves can be identified in the arms and measure approximately 0.4 mm in diameter.

The suckers show a developed infundibulum and a *Vampyroteuthis*-like attachment that extends into the acetabular cavity. Additionally, some suckers are encircled by what appear to be small, ovoid holes in the soft tissue. There is no direct evidence to suggest that these are related morphologically with remnants of sucker lining seen in some decabrachians, rather we conclude that these are a result of degraded epithelial tissue.

**MNHN.B.74244**

This specimen is fossilized in dorsal view (Supplementary Fig. 1), heavily compacted, and the body is not as well preserved as the holotype. The mantle is posteriorly rounded, and measures ~46 mm in composite length. As with the holotype, the CT scan indicates the presence of tissue laterally surrounding the body. Two dense, ovoid structures are located at the posterior-most area of the body and appear to be enveloped by soft tissue. These measure ~5 mm at their longest transects. These are situated in a similar location to the luminous organs in the extant *V. infernalis*, though are much larger in size. This is the only specimen in which these structures are observed. It is not possible to say if they are luminous organs, though it is likely that they represent portions of displaced cartilage or tissue. There is no evidence of an ink sac, and it is not possible to observe a gladius.

The massive head is short and the two spherical eyes (~6 - 7 mm in diameter) are compacted and preserved on offset lateral planes. No neck is evident.

The arms have undergone rotation prior to fossilization and are positioned in a lateral view. As with each of the specimens, arm pair I, the longest of all the arms (~43 – 51 mm), are situated in the dorsal position, and their axial nerves are clearly visible in CT imagery (Fig. 2). The cirri and suckers display the same configuration as the other two specimens. On the dorsal arms, the cirri diameter tapers distally (~2 – 1 mm) and the two distal suckers (~2 mm diameter) have an elongated attachment like that of the holotype. In profile view it is possible to see that the distal end of the attachment extends into the acetabular chamber. No sucker lining is visible.

The other three arm pairs (III-V) are shorter in length (~24-36 mm) and lie more ventrally. Of the redescribed specimens, MNHN.B.74244 has the best-preserved armature. Each arm has a single row of suckers (up to 10 are visible per row) that run the length of the arm. These suckers also have a *Vampyroteuthis*-like attachment that extends into the acetabulum (Fig. 2). The suckers are framed by paired cirri, the diameter of which (suckers and cirri) is < 2mm. These both taper towards the tips of the arms. There is no evidence of a pair of retractable filaments (arm pair II) like those seen in *V. infernalis*.

**MNHN.B.74243:**

This specimen is the most poorly preserved of the three and considerable portions of the body and arm crown are missing. Fossilized in lateral view (Supplementary Fig. 1) it exhibits a tapered, fusiform shape and has a mantle length of ~46 mm.

As in the other two specimens, there is no internal organ that suggests that an ink sac was present, nor evidence of luminous organs. The extent of fins is particularly difficult to determine in this specimen. Posterior bulges, representative of internal fin cartilage, are present, though the CT scan shows these structures are distorted. Fin soft tissue is either not well retained or fossilized in such a way that it does not extend from the body. Soft tissue is present under the eye and terminates just in front of it. Fischer & Riou^1^ suggested this was a long, muscular funnel, though we cannot confirm if this is funnel or mantle tissue.

It is apparent that the head has undergone considerable oblique twisting and compaction as the eyes are located almost on top of each other. They range in diameter from ~5 - 6 mm.

The dorsal arm pair (~53-57 mm) on MNHN.B.74243 are the longest of the three and have the same sucker and cirri configuration as the other two specimens. Some of the arm ornamentation is lost, though it is possible to make out a repetitive slight thickening pattern on the distal arm that corresponds with the basal parts of the cirri on the other two specimens.

Arm pairs III-V are shorter than the dorsal pair and are ~31-32 mm in length. As with the other two specimens, there is nothing to suggest there are any retractable filaments (arm pair II in *V. infernalis*). As it is not possible to identify the tips of the intermediate arms, they could possibly be longer. Many of the cirri and suckers on these arms are not preserved are not clearly able to be segmented. The exception to this is the most ventral arm pair, where the distal section displays a well-preserved sucker row flanked by cirri. The diameter of the few suckers that are measurable range between ~1 and ~2 mm. The smaller suckers are most distal. As with the holotype, there appear to be small holes surrounding some of the suckers.

1. **Measurements and Ratios**

All measurements below are composite (following the contour of the element measured if possible) and based on what is preserved. Each element has potentially undergone compaction and/or distortion prior to fossilization. Measurements are listed in mm unless otherwise noted.

**Fossil Material:**

**Supplementary Table S1: Overall measurements for the 3 *V. rhodanica* specimens (**MNHN.B.74247; MNHN.B.74244; MNHN.B.74243)

|  | MNHN.B.74247 | MNHN.B.74244 | MNHN.B.74243 |
| --- | --- | --- | --- |
| Dorsal arm length (longest) | 51.2 | 50.5 | 57.2 |
| Dorsal arm length (Shortest) | 48.5 | 42.8 | 53.4 |
| Sessile arm length (Longest) | 33.5 | 35.9 | 32 |
| Sessile arm length (Shortest) | 30.7 | 23.6 | 30.5 |
| Mantle (body) length (mid eye to posterior) | 43 | 46 | 45.6 |
| Total body length (body + longest dorsal arms) | 94.2 | 96.5 | 102.6 |
| Total body length (body + longest sessile arms) | 76.3 | 82 | 77.4 |
| Head width (widest part) | 24.6 | 24 | 23.3 |
| Sucker Diameter - dorsal (largest) | 2.4 | 2.2 | 1.8 |
| Sucker Diameter – dorsal (smallest) | 1.9 | 2.1 | 1.5 |
| Sucker Diameter – Sessile (largest) | 1.7 | 1.6 | 1.7 |
| Sucker Diameter – sessile (smallest) | 0.8 | 0.8 | 0.7 |
| Infundibulum width (max) |  | 0.49 |  |
| Acetabular cavity width (max) |  |  |  |
| Cirri height - sessile (longest) | 1.6 | 1.8 | N/A |
| Cirri height - sessile (Shortest) | 0.5 | 0.9 | N/A |
| Cirri diameter - sessile (largest) | 1.8 | 1.8 | 0.8 |
| Cirri diameter - sessile (smallest) | 0.8 | 1 | 1 |
| Cirri height - dorsal (longest) | 1 | 2.2 | 1.1 |
| Cirri height - dorsal (Shortest) | 3.2 | N/A | 0.8 |
| Cirri diameter - dorsal (largest) | 0.8 | 1.6 | 0.8 |
| Cirri diameter - dorsal (smallest) | 1.7 | 0.7 | 0.4 |

**Supplementary Table S2: Sample Arm (Arm pair V) measurements: *V. rhodanica* (MNHN.B.74244)**

| **Sessile Arm** | **Proximal** | | |  | | |  | | |  | |  | | |  | | |  | |  | | |  | | | **Distal** | | |
| --- | --- | --- | --- | --- | --- | --- | --- | --- | --- | --- | --- | --- | --- | --- | --- | --- | --- | --- | --- | --- | --- | --- | --- | --- | --- | --- | --- | --- |
| Sucker Diameter | 1.5 | | | 1.5 | | | N/A | | | 1.4 | | 1.5 | | | 1.2 | | | 1.0 | | 1.0 | | | 0.7 | | | 0.6 | | |
| Distance btwn suckers | | | 0.9 | | N/A | | | N/A | | | 0.7 | | | 0.6 | | 0.6 | | | 0.7 | | 0.5 | | | | 0.5 | |  | |
| Cirri diameter | 1.4 | | | 1.7 | | | 1.4 | | | 1.6 | | 1.2 | | | 1.4 | | | 0.9 | | 0.9 | | | 0.8 | | | 0.6 | | |
| Distance btwn cirri | | 0.2 | | | | 0.3 | | | 0.5 | | 0.7 | | 0.7 | | | | 0.7 | | 0.9 | | | 0.7 | | 0.6 | | | |  |
| Infundibulum width | 0.4 | | | 0.5 | | | N/A | | | 0.4 | | 0.5 | | | 0.4 | | | 0.3 | | 0.3 | | | N/A | | | N/A | | |
| Acetabular cavity width | 0.7 | | | 1.0 | | | N/A | | | 0.5 | | 0.4 | | | 0.5 | | | 0.5 | | N/A | | | N/A | | | N/A | | |
| Sphincter distance | N/A | | | N/A | | | N/A | | | 0.5 | | 0.4 | | | 0.5 | | | 0.5 | | N/A | | | N/A | | | N/A | | |
| Sucker height (external) | 0.7 | | | 0.7 | | | N/A | | | 0.8 | | 0.7 | | | 0.5 | | | 0.5 | | N/A | | | N/A | | | N/A | | |

**Supplementary Table S3a: Sample Arm (Arm Pair I) sucker measurements: *V. rhodanica* (MNHN.B.74244)**

| **Dorsal Arms (Sucker Measurements)** | **Arm A** | **Arm A** | | **Arm B** | | **Arm B** |
| --- | --- | --- | --- | --- | --- | --- |
|  | **Proximal** | **Distal** | | **Proximal** | | **Distal** |
| **Attachment diameter (base)** | 0.7 | 0.5 | | 0.6 | | 0.5 |
| **Attachment diameter (top)** | 0.6 | 0.6 | | 0.6 | | 0.5 |
| **Attachment diameter (below acetabulum)** | 0.5 | 0.4 | | 0.5 | | 0.4 |
| **Attachment length** | 0.9 | 0.7 | | 0.7 | | 0.7 |
| **Amount of attachment in acetabulum** | 0.5 | 0.5 | | 0.4 | | 0.4 |
| **Infundibulum diameter** | 0.9 | 0.5 | | 0.7 | | 0.7 |
| **Acetabular cavity diameter** | 1.0 | 0.8 | | 0.8 | | 0.9 |
| **Distance btwn sphincter** | 0.4 | 0.5 | | 0.6 | | 0.6 |
| **Sucker height (external)** | 0.9 | 0.9 | | 0.8 | | 0.9 |
| **Sucker height (internal)** | 0.7 | 0.7 | | 0.6 | | 0.6 |
| **Acetabular cavity base to sphincter** | 0.4 | 0.2 | | 0.4 | | 0.3 |
| **Sucker diameter** | 2.4 | 2.3 | | 2.1 | | 1.9 |
| **Distance between suckers (at attachment base)** |  | 1.1 |  | | 1.5 |  |

**Supplementary Table S3b: Sample Arm (Arm Pair I) cirri measurements: *V. rhodanica* (MNHN.B.74244)**

| **Dorsal Arm Cirri: Diameter at base** | **Proximal** | |  |  |  |  | **Distal** |
| --- | --- | --- | --- | --- | --- | --- | --- |
| **A** | 1.8 | 1.7 | | 1.2 | 1.1 | 1.0 | N/A |
| **B** | 1.1 | 1.2 | | 1.1 | 1.5 | N/A | N/A |

**Extant Material:**

**Supplementary Table S4a: Sample arm (Arm Pair IV) sucker measurements: *V. infernalis* (AMNH IZC 361496)**

| **Sample Arm (Suckers):** | **Sucker 1 (Proximal)** | **Sucker 2** | **Sucker 3** | **Sucker 4** | **Sucker 5 (Distal)** |
| --- | --- | --- | --- | --- | --- |
| Infundibulum width | 0.19 | 0.17 | 0.19 | 0.17 | 0.21 |
| Acetabular cavity width | 0.35 | 0.20 | 0.22 | 0.27 | 0.25 |
| Sphincter distance | 0.18 | 0.20 | 0.25 | 0.12 | 0.14 |
| Sucker diameter | 0.67 | 0.66 | 0.54 | 0.61 | 0.57 |

**Highlighted sucker measurements used in the comparison

**Supplementary Table S4b: Sample arm (Arm Pair IV) cirri measurements: *V. infernalis* (AMNH IZC 361496)**

| **Sample Arm (Cirri):** | **Prox.** |  |  |  |  |  |  |  |  |  |  |  |  | **Dist.** |
| --- | --- | --- | --- | --- | --- | --- | --- | --- | --- | --- | --- | --- | --- | --- |
| Cirri diameter at base | 0.4 | 0.5 | 0.5 | 0.6 | 0.7 | 0.7 | 0.7 | 0.6 | 0.7 | 0.6 | 0.5 | 0.4 | 0.5 | 0.6 |
| Distance btwn cirri | 1.9 | 1.7 | 1.4 | 1.1 | 1.1 | 0.8 | 0.7 | 0.4 | 0.6 | 0.6 | 0.5 | N/A | N/A | N/A |

**Supplementary Table S4c: Sample arm (Arm Pair IV) sucker measurements: *V. infernalis* (YPM IZ 18279.GP)**

| **Sample Arm:** | **Sucker 1 (Proximal)** | **Sucker 2** | **Sucker 3** | **Sucker 4** | **Sucker 5 (Distal)** |
| --- | --- | --- | --- | --- | --- |
| Infundibulum width | 0.10 | 0.96 | 0.07 | 0.13 | 0.14 |
| Acetabular cavity width | 0.26 | 0.20 | 0.25 | 0.54 | 0.26 |
| Sphincter distance | 0.13 | 0.05 | 0.13 | 0.26 | 0.19 |
| Sucker diameter | 0.46 | 0.37 | 0.4 | 0.78 | 0.53 |

**Highlighted sucker measurements used in the comparison

**Supplementary Table S5a: Ratios based on the composite measurements of *V. rhodanica* (this study), and mean values of *V. infernalis* from Pickford 1949**^2^**.**

|  | ***V. rhodanica* MNHN.B.74247** | ***V. rhodanica* MNHN.B.74244** | ***V. rhodanica* MNHN.B.74243** | ***V. infernalis* (Pickford**^2^**) Mean** |
| --- | --- | --- | --- | --- |
| **Absolute Size** | 43 | 46 | 46 | 49.2 (male);  59.5 (female) |
| **Head Width Index** | 0.56 | 0.49 | 0.51 | 0.94 |
| **Length of Longest Arm (Dorsal)** | 54.4 | 52.3 | 55.6 |  |
| **Length of Longest Arm (Sessile)** | 43.9 | 43.8 | 41.3 | 63 |
| **Longest:shortest arm (Dorsal)** | 59.9 | 46.7 | 53.3 |  |
| **Longest:shortest arm (Sessile)** | 91.6 | 65.7 | 95.3 | 72 |
| **Sucker diameter index (Dorsal)** | 5.6 | 4.8 | 3.9 |  |
| **Sucker diameter index (Sessile)** | 3.9 | 3.5 | 3.7 | 3.7 |
| **Cirri length index (Dorsal)** | 2.3 | 5.2 | 2.4 |  |
| **Cirri length index (Sessile)** | 3.7 | 3.9 | N/A | 8.5 |
| **Arm:Mantle ratio (Dorsal)** | 0.8 | 0.9 | 0.8 |  |
| **Arm:Mantle ratio (Sessile)** | 0.8 | 0.8 | 0.7 |  |

The mean ratios for *V. infernalis* were taken directly from Pickford^2^. This table indicates that the head width of *V. rhodanica* is ~50% the length of the body, whereas the head width of *V. infernalis* is ~94%. See below for the ratio equations used. As *V. infernalis* does not have an elongated Arm Pair 1, the ratios were compared with the sessile arms in *V. rhodanica*. Ratios for the infundibulum diameter and cirri diameter were not listed in Pickford^2^, so the ratio equations shown in Table 5b were utilised:

**Supplementary Table S5b: Ratios based on the composite measurements of *V. rhodanica* (MNHN.B.74244) and *V. infernalis* (YPM IZ 18279.GP, and AMNH IZC 361496). Ratios were adapted from Pickford (1949)**^2^

| **Sucker ratios:** | **MNHN.B.74244** | **YPM IZ 18279.GP** | **AMNH IZC 361496** | **Ratio Equations** |
| --- | --- | --- | --- | --- |
| Infundibulum size ratio | 31 | 17 | 13 | Largest measurement x 100/sucker diameter |
| Cirri diameter ratio | 3.5 | N/A | 1.9 | Largest measurement x 100/mantle length |

**Ratios:**

Absolute Size (*Mantle Length*):

*V. infernalis* sizes were taken from Pickford^2^. In that sample, the mean size was 49.2 (male), 59.5 (female). The range was 44 – 55 (male) (4 specimens) and 55 – 63 (female) (two specimens). The 3 *V. rhodanica* specimens MNHN.B.74247 (~43), MNHN.B.74244 (~46), and MNHN.B.74243 (~46) are consistent with the size range for individual male specimens of *V. infernalis*.

Head width index

*V. rhodanica* has a proportionally smaller head width than *V. infernalis*. This is expected as *V. infernalis* was described as having a wider head and a shorter mantle than *V. rhodanica* by Fischer & Riou^1^. The 3 MNHN specimens of *V. rhodanica* have a head width that is ~49 – 56% the length of the body. The range of head width ratios of *V. infernalis* described in Pickford^2^ is 76 – 112%. The average head width is ~94% the length of the mantle. The head width of *V. rhodanica* is roughly half the mantle length.

*Head Width X 100/Mantle Length*

Length of longest arm

Pickford^2^ noted two equations for this index, the arm length index, and the mantle length index. This study followed Pickford and used the arm length index for the *V. rhodanica* specimens. *V. infernalis* lacks the longer dorsal arm pair seen in *V. rhodanica*. While this index was calculated for both the dorsal arms, and arm pairs III-V in *V. rhodanica*, the ratios listed in Supplementary Table S5a utilized just arm pairs III – V.

When including the dorsal arms, the index for our *V. rhodanica* specimens range from ~52 – 56%. Using just the lengths of the sessile arms, the range is ~41 – 44%. Pickford^2^ recorded that in *V. infernalis*, the mean value of the index was 63%, and ranged from 51 – 77%.

*Arm length index: Length of longest arm x 100/ total length*

*Mantle length X 100/longest arm*

Difference between longest and shortest arms:

This index shows the limits of variability in the arm crown. As for the Arm Length Index, calculations were performed on both the dorsal (Arm Pair I) and non-dorsal arms (arm pairs III – V), though only the non-dorsal arms are included in Supplementary Table S5a.

In the arm crown of *V. rhodanica*, the shortest sessile arms were roughly half the size of the longest dorsal arms (~47 – 60%). A comparison of just the non-dorsal arms showed less variability with both the MNHN.B.74247, and MNHN.B.74243 expressing a range in the 90s (92, and 95% respectively). The index for specimen 74244 was 66%. This difference may reflect loss of length in arm pairs III-V. The mean index for *V. infernalis* is 72%. This is expected as the arm crown of *V. infernalis* shows less variability in dorsal arm length. The range, however, is wide and falls between 54 – 92%.

*Length of the shortest arm x 100/length of longest arm*

Sucker diameter index

This index reflects allometry with size. Again, this index was calculated both including, and excluding the dorsal arms. The range for *V. infernalis* is 2.0 – 4.8 % with a mean of 3.7%; this is consistent with the index returned for the sessile arms only (3.4 – 3.9%). Suckers on the dorsal arms of *V. rhodanica* have a slightly higher index and range from 3.9 – 5.6%.

*Diameter of largest sucker x 100/ mantle-length*

**III) Microtomography**

The three *V. rhodanica* fossils were initially imaged using X-ray Computed Tomography (µCT) at the AST-RX platform at the Muséum National d’Histoire Naturelle, Paris (MNHN, Paris, France), and then at the European Synchrotron Radiation Facility (ESRF, Grenoble, France). Acquisition at the AST-RX platform generated data with a voxel size of 88.60 µm. At the ESRF, the experiment was done at the ID19 beamline using propagation phase-contrast X-ray synchrotron microtomography (PPC-SRµCT). The experimental setup consisted of a filtered pink beam (Wiggler W150B gap: 55 mm; see Table S6 for filters) with detected total integrated energy ranging from 112 keV to 136.3 keV depending on the filter used; a sample-detector propagation distance of 16 m; and an indirect detector comprising a 500 mm LuAG:Ce crystal scintillator, a set of Hasselblad photographic lenses (Victor Hasselblad AB, Gothenburg, Sweden) providing a 1x magnification and a FReLoN-2k camera, generating data with an isotropic voxel size of 12.64 µm. Given the limited size of the X-ray beam with this setup (7.58 mm vertically and 25.89 mm horizontally), several acquisitions were necessary to cover the specimens on the vertical axis with the motorised sample manipulator, keeping a 50% overlap between consecutive acquisitions. To overcome the limitation horizontally, the centre of rotation of the sample stage was set near the edge of the recorded projections, allowing to almost double the field of view in reconstructed data (i.e., so called half-acquisition^3^). For all specimens, each acquisition consisted of 4998 projections of 0.1 second exposure each, over a 360° rotation. The tomographic reconstruction was done with the PyHST2 software^4^ using the single distance phase retrieval approach^5^ generating stack of 32-bits files. Follow up post-processing included: ring corrections^6^; change of the dynamic range from 32-bit to 16-bit; vertical concatenation of the dataset using a weighted averaging; export of the concatenated dataset as stack of 16-bit tiff files.

**Supplementary Table S6: List of parameters used for propagation phase-contrast X-ray synchrotron microtomography at the ID19 beamline of the ESRF.**

| Specimen | ID | gap | Filters | energy | ODD | voxel size | exposure | projection |
| --- | --- | --- | --- | --- | --- | --- | --- | --- |
| MNHN.B.74247 | W150B | 55 mm | W: 0.5 mm Cu: 6 mm Al: 2.8 mm | 136.3 keV | 16 m | 12.64 µm | 0.1 s | 4998 |
| MNHN.B.74243 | W150B | 55 mm | W: 0.5 mm Cu: 6 mm Al: 2.8 mm | 136.3 keV | 16 m | 12.64 µm | 0.1 s | 4998 |
| MNHN.B.74244 | W150B | 55 mm | Cu: 8 mm Al 2.8 mm | 112 keV | 16 m | 12.64 µm | 0.1 s | 4998 |

Imaging of extant *V. infernalis* were done using µCT at the Microscopy and Imaging Facility of the American Museum of Natural History (New York, USA). The resulting voxel size were 38.40 µm (AMNH IZC 361496) and 18.25 µm (YPM IZ18279.GP). Both specimens were stained in a 1% PTA solution prior to scanning.

Final CT data were reduced in size using ImageJ software (cropping and binning 2x2x2), and then segmented using Mimics software (Materialise NV, Belgium, Version 21.0). The contrasting densities of the mineralized soft tissues were utilized to identify anatomical features for segmentation. Morphological reconstructions were carried out for the three *V. rhodanica* specimens incorporating all possible internal and external soft tissues. A full reconstruction of *V. infernalis* was carried out on AMNH IZC 361496. Some sucker tissues in YPM IZ18279.GP had more clearly defined boundaries and these were integrated into the analysis to augment the data gathered from AMNH IZC 361496.

1. Fischer, J.-C. & Riou, B. *Vampyronassa rhodanica nov. gen. nov sp*., vampyromorphe (Cephalopoda, Coleoidea) du Callovien inférieur de La Voulte-sur-Rhône (Ardèche, France). *Ann. de Paléontol.* **88**, 1–17 (2002).

2. Pickford, G. E. *Vampyroteuthis infernalis* Chun-An archaic dibranchiate cephalopod. II. External anatomy. *Dana Rep.* **32**, 1–132 (1949).

3. Carlson, K. J. *et al.* The endocast of MH1, *Australopithecus sediba*. *Science* **333**, 1402–1407 (2011).

4. Mirone, A., Brun, E., Gouillart, E., Tafforeau, P. & Kieffer, J. The PyHST2 hybrid distributed code for high speed tomographic reconstruction with iterative reconstruction and a priori knowledge capabilities. *Nuclear Instruments and Methods in Physics Research Section B: Beam Interactions with Materials and Atoms* **324**, 41–48 (2014).

5. Paganin, D., Mayo, S. C., Gureyev, T. E., Miller, P. R. & Wilkins, S. W. Simultaneous phase and amplitude extraction from a single defocused image of a homogeneous object. *Journal of microscopy* **206**, 33–40 (2002).

6. Lyckegaard, A., Johnson, G. & Tafforeau, P. Correction of ring artifacts in X-ray tomographic images. *Int. J. Tomo. Stat* **18**, 1–9 (2011).

7. Fuchs, D., Hoffmann, R. & Klug, C. Evolutionary development of the cephalopod arm armature: a review. *Swiss J. Palaeontol.* **140**, 1–18 (2021).

8. Young, R. E. & Vecchione, M. Analysis of morphology to determine primary sister-taxon relationships within coleoid cephalopods. *Am. Malacol. Bull.* **12**, 91–112 (1996).

9. Kruta, I. *et al.* *Proteroctopus ribeti* in coleoid evolution. *Palaeontology* **59**, 767–773 (2016).

10. Sutton, M., Perales-Raya, C. & Gilbert, I. A phylogeny of fossil and living neocoleoid cephalopods. *Cladistics* **32**, 297–307 (2016).

11. Goloboff, P. A., Farris, J. S. & Nixon, K. C. TNT, a free program for phylogenetic analysis. *Cladistics* **24**, 774–786 (2008).


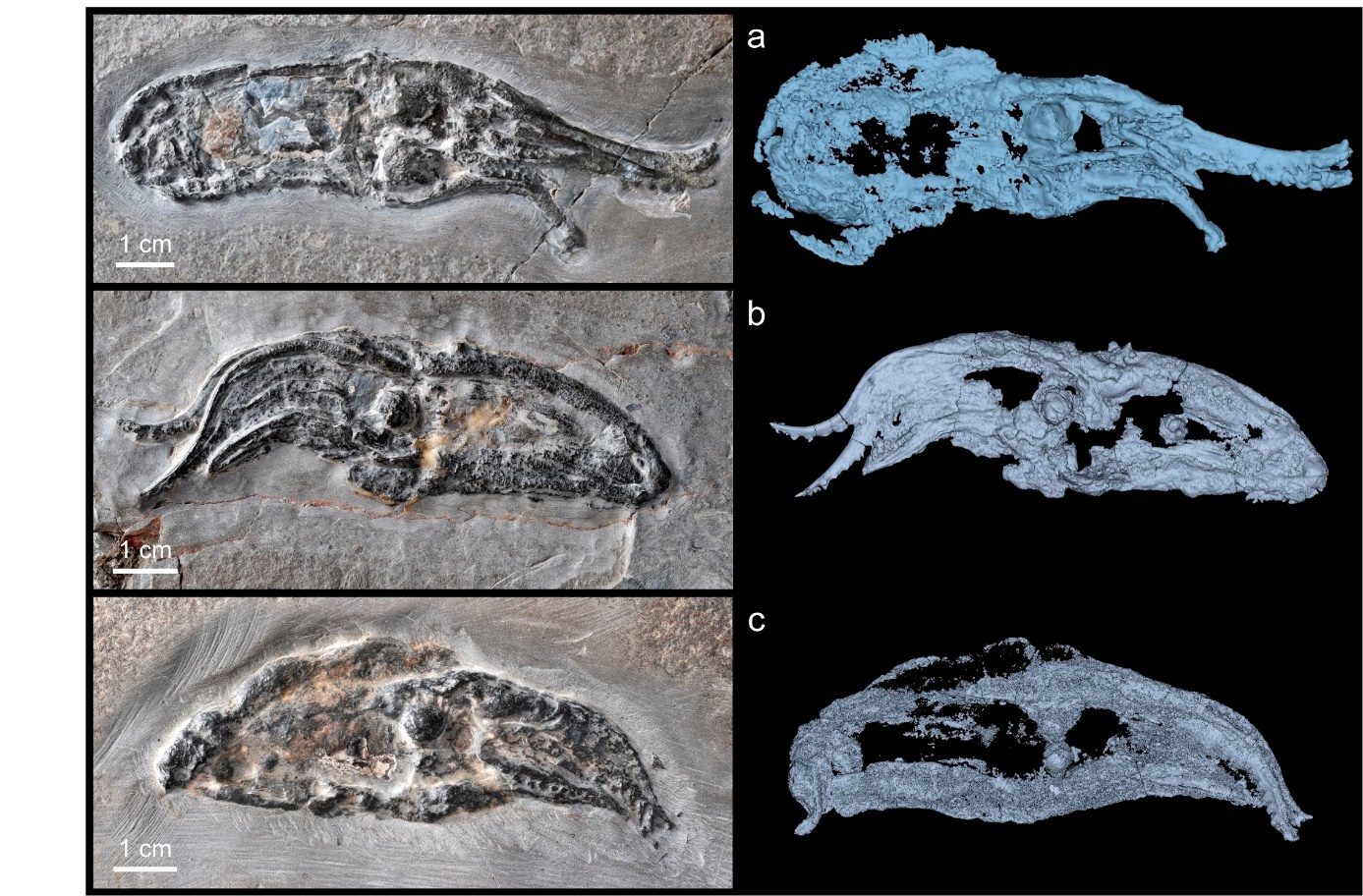


Supplementary Fig. 1. Photographs *(left)* by P. Loubry (CR2P), and 3D reconstructions *(right)* of the 3 *V. rhodanica* specimens. (**a**) MNHN.B.74244 (AST-RX). (**b**) MNHN.B.74243 (PPC-SR-µCT, ESRF). (**c**) MNHN.B.74247 (PPC-SR-µCT, ESRF). All reconstructions were created using Mimics software (Materialise NV, Belgium, Version 21.0).


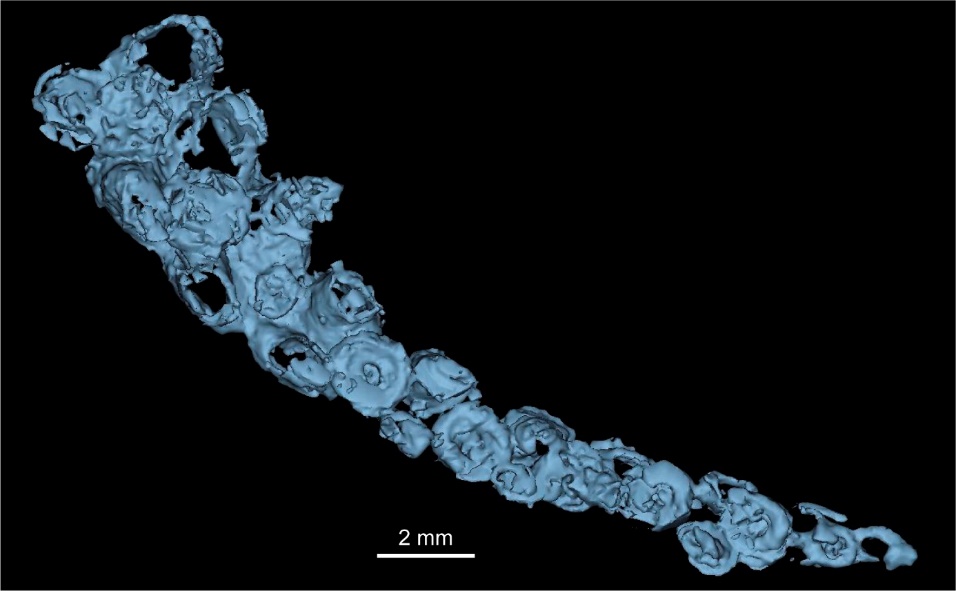


Supplementary Fig. 2. 3D reconstruction of a sessile arm (from arm pair V) of *V. rhodanica* MNHN.B.74244 showing the uniserial sucker row flanked by paired cirri. (PPC-SR-µCT, ESRF).


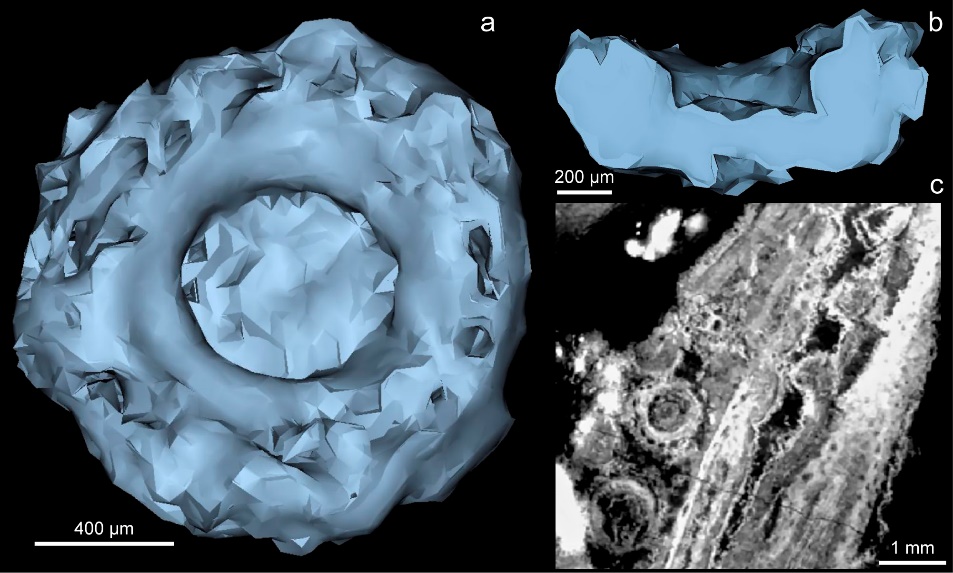


Supplementary Fig. 3. 3D Reconstruction of a sucker of MNHN.B.74243 showing the oval-shaped depressions. (**a**) Oral view showing the depressions encircling the outer perimeter of the sucker. (**b**) Profile view. (**c**) Slice (PPC-SR-µCT, ESRF) of the same sucker.


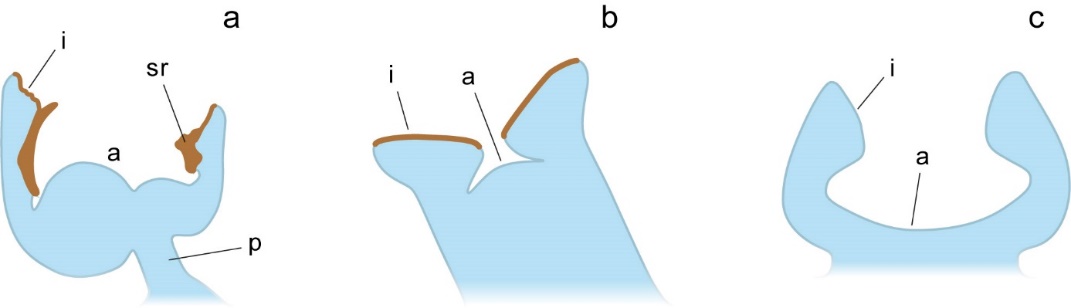


Supplementary Fig. 4. Simplified illustrations of the sucker and attachment types (in profile) of (**a**) Decabrachia, (**b**) Octobrachia, (**c**) Vampyromorpha. (**a,b**) adapted from Fuchs, Hoffmann, & Klug^7^. (**c**) adapted from Young & Vecchione^8^. (i) infundibulum, (a) acetabulum, (sr) sucker ring, (p) muscular, piston-like attachment.


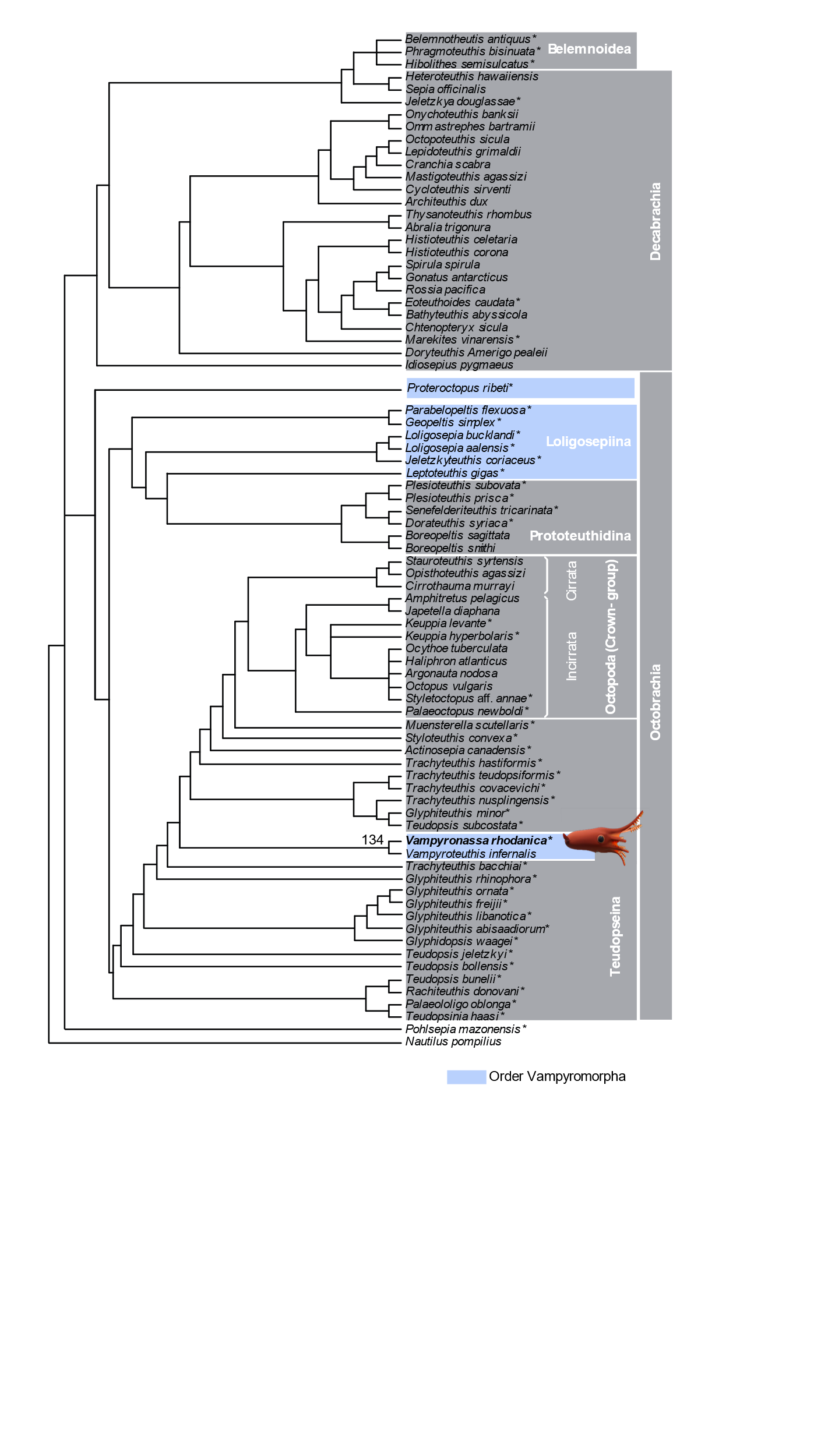


Supplementary Fig.5: Consensus tree obtained from 34 most parsimonious trees showing the relationship between *Vampyroteuthis infernalis* and *Vampyronassa rhodanica*. The matrix was modified from Kruta et al. ^9^, which was based on the matrix and parameters of Sutton et al.^10^ (2016). Data were analysed using TNT^11^. The state of character 90 (sucker stalks that are present but not clearly attached to the arm muscles) at node 134 were previously autapomorphic in *V. infernalis.* This study shows that this state is present in *V. rhodanica* and therefore synapomorphic.


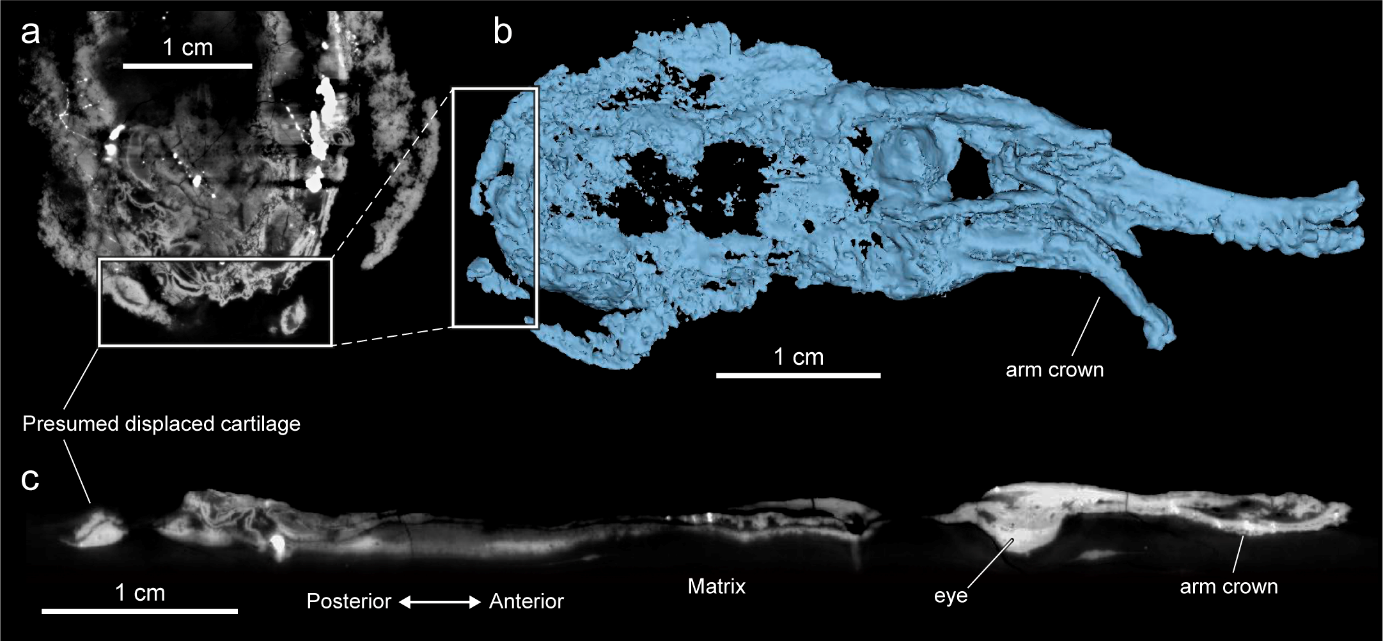


Supplementary Fig.6: **(a)** Axial slice (PPC-SR-µCT, ESRF) **(b)** 3D reconstruction and (**c**) sagittal slice (PPC-SR-µCT, ESRF) of MNHN.B.74244 showing the presumed displaced cartilage.
